# Poly[(μ_5_-5-amino­isophthalato)aqua­barium]

**DOI:** 10.1107/S1600536811037962

**Published:** 2011-09-30

**Authors:** Cheng-You Wu, Chia-Her Lin

**Affiliations:** a Department of Chemistry, Chung-Yuan Christian University, Chung-Li 320, Taiwan

## Abstract

In the title compound, [Ba(C_8_H_5_NO_4_)(H_2_O)]_*n*_, the Ba^II^ ion is eight-coordinated by six O atoms and one N atom from five 5-amino­isophthalate ligands and one water mol­ecule in a distorted dodeca­hedral geometry. The Ba^II^ ions are connected *via* the ligands into a layer parallel to (011). The layers are linked by N—H⋯O hydrogen bonds. The coordinated water mol­ecule is involved in intra­layer O—H⋯O hydrogen bonds.

## Related literature

For general background to metal coordination polymers, see: Kitagawa *et al.* (2004 ▶). For related structures, see: Kongshaug & Fjellvåg (2006 ▶); Wu & Lin (2010 ▶); Zeng *et al.* (2007 ▶).
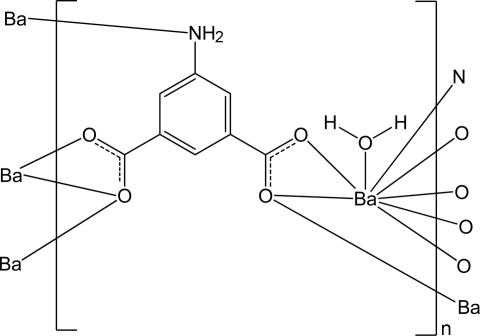


## Experimental

### 

#### Crystal data

[Ba(C_8_H_5_NO_4_)(H_2_O)]
*M* *_r_* = 334.48
Triclinic, 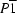

*a* = 7.7621 (1) Å
*b* = 7.9652 (1) Å
*c* = 8.3416 (1) Å
α = 79.618 (1)°
β = 65.574 (1)°
γ = 83.575 (1)°
*V* = 461.48 (1) Å^3^
*Z* = 2
Mo *K*α radiation
μ = 4.30 mm^−1^
*T* = 295 K
0.50 × 0.30 × 0.30 mm

#### Data collection

Bruker APEXII CCD diffractometer
Absorption correction: multi-scan (*SADABS*; Bruker, 2001 ▶) *T*_min_ = 0.232, *T*_max_ = 0.275
7950 measured reflections
2283 independent reflections
2230 reflections with *I* > 2σ(*I*)
*R*_int_ = 0.019

#### Refinement

*R*[*F* ^2^ > 2σ(*F* ^2^)] = 0.015
*wR*(*F* ^2^) = 0.040
*S* = 1.12
2283 reflections
136 parameters
H-atom parameters constrained
Δρ_max_ = 0.48 e Å^−3^
Δρ_min_ = −0.79 e Å^−3^

### 

Data collection: *APEX2* (Bruker, 2007 ▶); cell refinement: *SAINT* (Bruker, 2007 ▶); data reduction: *SAINT*; program(s) used to solve structure: *SHELXS97* (Sheldrick, 2008 ▶); program(s) used to refine structure: *SHELXL97* (Sheldrick, 2008 ▶); molecular graphics: *DIAMOND* (Brandenburg, 1999 ▶); software used to prepare material for publication: *SHELXTL* (Sheldrick, 2008 ▶).

## Supplementary Material

Crystal structure: contains datablock(s) I, global. DOI: 10.1107/S1600536811037962/hy2470sup1.cif

Structure factors: contains datablock(s) I. DOI: 10.1107/S1600536811037962/hy2470Isup2.hkl

Additional supplementary materials:  crystallographic information; 3D view; checkCIF report

## Figures and Tables

**Table 1:** Hydrogen-bond geometry (Å, °)

*D*—H⋯*A*	*D*—H	H⋯*A*	*D*⋯*A*	*D*—H⋯*A*
O1*W*—H1*WA*⋯O3^i^	0.89	1.90	2.770 (2)	165
O1*W*—H1*WB*⋯O2^ii^	0.83	1.95	2.770 (2)	167
N1—H1*A*⋯O2^iii^	0.96	2.16	3.067 (2)	157
N1—H1*B*⋯O4^iv^	0.99	2.19	3.176 (2)	175

Symmetry codes: (i) 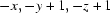; (ii) 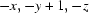; (iii) 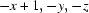; (iv) 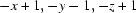.

**Fig. 1.**
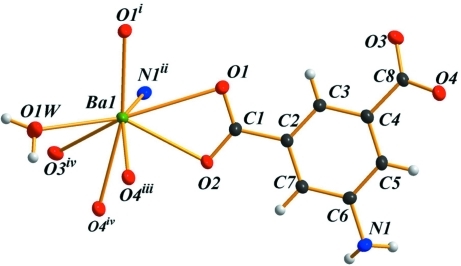
The asymmetric unit of the title compound. Displacement ellipsoids are drawn at the 50% probability level. [Symmetry codes: (i) -x, 1-y, 1-z; (ii) x, 1+y, z; (iii) -x, -y, 1-z; (iv) x, 1+y, -1+z.]

**Fig. 2.**
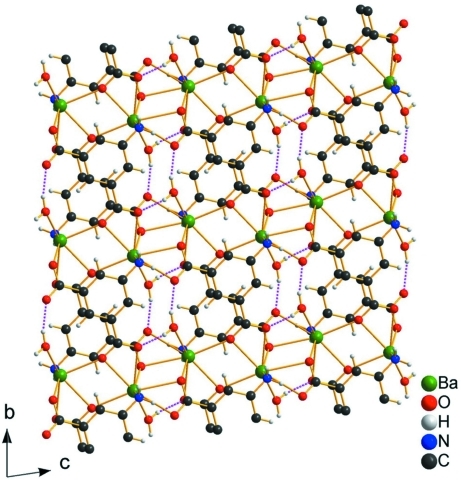
The layer structure of the title compound viewed along the a axis. Intralayer O—H···O hydrogen bonds are shown as pink dashed lines.

**Fig. 3.**
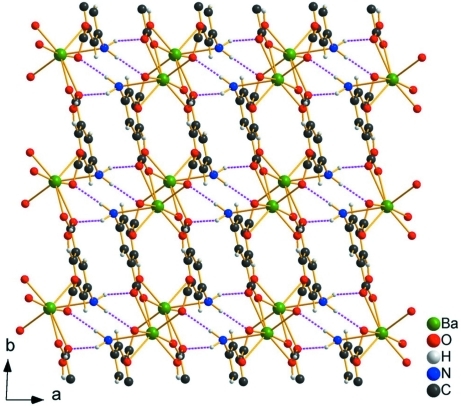
The crystal packing of the title compound viewed along the a axis. Interlayer N—H···O hydrogen bonds are shown as pink dashed lines.

## supplementary crystallographic information

### Comment

The increasingly rapid development of metal coordination polymers over the past two decades has attracted considerable attention due to their structural diversity and important applications (Kitagawa *et al.*, 2004). 5-Aminoisophthalic acid has been successively reported as sodium (Zeng *et al.*, 2007), zinc (Kongshaug & Fjellvåg, 2006) and magnesium complexes (Wu & Lin, 2010). In our continuous investigations in metal coordination polymers, we report here the structure of a new Ba(II) coordination polymer based on the 5-aminoisophthalate ligand.

In the title compound (Fig. 1), the Ba^II^ ion is eight-coordinated by six O atoms and one N atom from five 5-aminoisophthalate ligands and one water molecule in a distorted dodecahedral geometry. The Ba—O distances range from 2.6808 (16) to 2.8813 (17) Å. The Ba—N distance is 2.918 (2) Å. The BaO_7_N dodecahedra are connected *via* the anionic ligands into a layer parallel to ( 0 1 1). The coordinated water molecule is involved in intralayer O—H···O hydrogen bonds (Table 1 , Fig. 2). These layers are linked by interlayer N—H···O hydrogen bonds (Fig. 3).

### Experimental

Solvothermal reactions were carried out at 423 K for 2 d in a Teflon-lined acid digestion bomb with an internal volume of 23 ml followed by slow cooling at 6 K h^-1^ to room temperature. A single-phase product consisting of transparent crystals was obtained from a mixture of 5-aminoisophthalic acid (C_8_H_7_NO_4_, 0.145 g, 0.8 mmol), Ba(NO_3_)_2_.4H_2_O (0.105 g, 0.2 mmol), methanol (5.0 ml) and H_2_O (1.0 ml).

### Refinement

H atoms bound to C atoms were positioned geometrically, with C—H = 0.93 Å and *U*_iso_(H) = 1.2*U*_eq_(C). H atoms bound to N and O atoms were located in a difference Fourier map and fixed in refinements, with *U*_iso_(H) = 1.5*U*_eq_(O) or 1.2*U*_eq_(N).

### Crystal data

**Table:**

[Ba(C_8_H_5_NO_4_)(H_2_O)]	*Z* = 2
*M**_r_* = 334.48	*F*(000) = 316
Triclinic, *P*1	*D*_x_ = 2.407 Mg m^−^^3^
Hall symbol: -P 1	Mo *K*α radiation, λ = 0.71073 Å
*a* = 7.7621 (1) Å	Cell parameters from 120 reflections
*b* = 7.9652 (1) Å	θ = 2.6–31.8°
*c* = 8.3416 (1) Å	µ = 4.30 mm^−^^1^
α = 79.618 (1)°	*T* = 295 K
β = 65.574 (1)°	Block, colourless
γ = 83.575 (1)°	0.50 × 0.30 × 0.30 mm
*V* = 461.48 (1) Å^3^	

### Data collection

**Table:**

Bruker APEXII CCD diffractometer	2283 independent reflections
Radiation source: fine-focus sealed tube	2230 reflections with *I* > 2σ(*I*)
graphite	*R*_int_ = 0.019
Detector resolution: 8.3333 pixels mm^-1^	θ_max_ = 28.3°, θ_min_ = 2.6°
φ and ω scans	*h* = −10→10
Absorption correction: multi-scan (*SADABS*; Bruker, 2001)	*k* = −10→10
*T*_min_ = 0.232, *T*_max_ = 0.275	*l* = −11→10
7950 measured reflections	

### Refinement

**Table:**

Refinement on *F*^2^	Primary atom site location: structure-invariant direct methods
Least-squares matrix: full	Secondary atom site location: difference Fourier map
*R*[*F*^2^ > 2σ(*F*^2^)] = 0.015	Hydrogen site location: inferred from neighbouring sites
*wR*(*F*^2^) = 0.040	H-atom parameters constrained
*S* = 1.12	*w* = 1/[σ^2^(*F*_o_^2^) + (0.0203*P*)^2^ + 0.2964*P*] where *P* = (*F*_o_^2^ + 2*F*_c_^2^)/3
2283 reflections	(Δ/σ)_max_ = 0.001
136 parameters	Δρ_max_ = 0.48 e Å^−^^3^
0 restraints	Δρ_min_ = −0.79 e Å^−^^3^

### Fractional atomic coordinates and isotropic or equivalent isotropic displacement parameters (Å^2^)

**Table:**

	*x*	*y*	*z*	*U*_iso_*/*U*_eq_	
Ba1	0.047838 (16)	0.597922 (13)	0.212249 (13)	0.01817 (5)	
O1	0.1383 (2)	0.32074 (19)	0.4588 (2)	0.0281 (3)	
O1W	−0.1803 (3)	0.8279 (2)	0.0982 (2)	0.0345 (4)	
H1WA	−0.1919	0.9411	0.0794	0.052*	
H1WB	−0.2053	0.7847	0.0267	0.052*	
O2	0.2226 (3)	0.27828 (19)	0.18144 (19)	0.0286 (3)	
O3	0.2563 (3)	−0.1752 (2)	0.8942 (2)	0.0293 (3)	
O4	0.2032 (2)	−0.42403 (18)	0.84296 (19)	0.0243 (3)	
N1	0.4296 (3)	−0.3479 (2)	0.1704 (2)	0.0222 (3)	
H1A	0.5246	−0.2931	0.0622	0.027*	
H1B	0.5405	−0.4208	0.1747	0.027*	
C1	0.2029 (3)	0.2256 (2)	0.3391 (3)	0.0198 (4)	
C2	0.2556 (3)	0.0420 (2)	0.3867 (3)	0.0184 (4)	
C3	0.2367 (3)	−0.0217 (2)	0.5594 (3)	0.0204 (4)	
H3	0.1983	0.0512	0.6446	0.025*	
C4	0.2751 (3)	−0.1940 (2)	0.6051 (3)	0.0180 (4)	
C5	0.3385 (3)	−0.3012 (2)	0.4757 (3)	0.0191 (4)	
H5	0.3610	−0.4171	0.5071	0.023*	
C6	0.3688 (3)	−0.2373 (2)	0.2996 (3)	0.0179 (4)	
C7	0.3232 (3)	−0.0654 (3)	0.2571 (3)	0.0193 (4)	
H7	0.3382	−0.0221	0.1411	0.023*	
C8	0.2440 (3)	−0.2676 (3)	0.7936 (3)	0.0191 (4)	

### Atomic displacement parameters (Å^2^)

**Table:**

	*U*^11^	*U*^22^	*U*^33^	*U*^12^	*U*^13^	*U*^23^
Ba1	0.02706 (8)	0.01489 (7)	0.01284 (7)	0.00289 (5)	−0.00903 (5)	−0.00259 (4)
O1	0.0457 (9)	0.0188 (7)	0.0174 (7)	0.0088 (6)	−0.0116 (7)	−0.0057 (6)
O1W	0.0527 (11)	0.0238 (8)	0.0411 (10)	0.0094 (7)	−0.0335 (9)	−0.0103 (7)
O2	0.0509 (10)	0.0182 (7)	0.0160 (7)	0.0084 (7)	−0.0156 (7)	−0.0020 (5)
O3	0.0511 (10)	0.0211 (7)	0.0205 (7)	−0.0045 (7)	−0.0190 (7)	−0.0013 (6)
O4	0.0381 (8)	0.0156 (7)	0.0187 (7)	−0.0021 (6)	−0.0125 (6)	0.0016 (5)
N1	0.0284 (9)	0.0191 (8)	0.0181 (8)	0.0046 (7)	−0.0081 (7)	−0.0065 (6)
C1	0.0266 (10)	0.0154 (9)	0.0153 (9)	0.0024 (7)	−0.0072 (8)	−0.0021 (7)
C2	0.0232 (9)	0.0162 (9)	0.0150 (9)	0.0025 (7)	−0.0078 (7)	−0.0018 (7)
C3	0.0298 (10)	0.0159 (9)	0.0145 (8)	0.0018 (7)	−0.0085 (8)	−0.0022 (7)
C4	0.0211 (9)	0.0177 (9)	0.0153 (8)	−0.0002 (7)	−0.0085 (7)	−0.0007 (7)
C5	0.0237 (9)	0.0145 (8)	0.0181 (9)	0.0023 (7)	−0.0090 (8)	−0.0005 (7)
C6	0.0193 (9)	0.0178 (9)	0.0165 (9)	0.0019 (7)	−0.0070 (7)	−0.0047 (7)
C7	0.0250 (9)	0.0173 (9)	0.0143 (8)	0.0022 (7)	−0.0078 (7)	−0.0012 (7)
C8	0.0244 (9)	0.0176 (9)	0.0149 (9)	0.0018 (7)	−0.0091 (7)	0.0001 (7)

### Geometric parameters (Å, °)

**Table:**

Ba1—O1^i^	2.6815 (15)	N1—C6	1.410 (2)
Ba1—O1W	2.7266 (16)	N1—H1A	0.9638
Ba1—O4^ii^	2.7392 (16)	N1—H1B	0.9930
Ba1—O2	2.7502 (15)	C1—C2	1.502 (3)
Ba1—O4^iii^	2.8371 (15)	C2—C3	1.390 (3)
Ba1—O3^iii^	2.8807 (16)	C2—C7	1.393 (3)
Ba1—N1^iv^	2.9094 (19)	C3—C4	1.390 (3)
Ba1—O1	2.9675 (15)	C3—H3	0.9300
O1—C1	1.263 (2)	C4—C5	1.391 (3)
O1W—H1WA	0.8880	C4—C8	1.504 (3)
O1W—H1WB	0.8346	C5—C6	1.394 (3)
O2—C1	1.256 (2)	C5—H5	0.9300
O3—C8	1.249 (2)	C6—C7	1.395 (3)
O4—C8	1.272 (2)	C7—H7	0.9300
			
O1^i^—Ba1—O1W	89.50 (5)	O2—Ba1—Ba1^v^	72.77 (3)
O1^i^—Ba1—O4^ii^	105.30 (5)	O4^iii^—Ba1—Ba1^v^	36.03 (3)
O1W—Ba1—O4^ii^	71.13 (5)	O3^iii^—Ba1—Ba1^v^	76.62 (3)
O1^i^—Ba1—O2	117.46 (4)	N1^iv^—Ba1—Ba1^v^	120.53 (3)
O1W—Ba1—O2	143.56 (5)	O1—Ba1—Ba1^v^	112.75 (3)
O4^ii^—Ba1—O2	77.97 (5)	C8^iii^—Ba1—Ba1^v^	54.33 (4)
O1^i^—Ba1—O4^iii^	167.63 (5)	C1—Ba1—Ba1^v^	91.86 (4)
O1W—Ba1—O4^iii^	78.43 (5)	C6^iv^—Ba1—Ba1^v^	143.55 (3)
O4^ii^—Ba1—O4^iii^	73.57 (5)	C1—O1—Ba1^i^	157.18 (14)
O2—Ba1—O4^iii^	74.63 (4)	C1—O1—Ba1	90.64 (12)
O1^i^—Ba1—O3^iii^	126.70 (5)	Ba1^i^—O1—Ba1	106.97 (5)
O1W—Ba1—O3^iii^	67.04 (5)	Ba1—O1W—H1WA	134.8
O4^ii^—Ba1—O3^iii^	110.30 (4)	Ba1—O1W—H1WB	107.5
O2—Ba1—O3^iii^	107.93 (5)	H1WA—O1W—H1WB	111.2
O4^iii^—Ba1—O3^iii^	45.63 (4)	C1—O2—Ba1	101.23 (12)
O1^i^—Ba1—N1^iv^	97.88 (5)	C8—O3—Ba1^vi^	93.35 (12)
O1W—Ba1—N1^iv^	126.35 (5)	C8—O4—Ba1^ii^	129.23 (13)
O4^ii^—Ba1—N1^iv^	151.39 (5)	C8—O4—Ba1^vi^	94.88 (12)
O2—Ba1—N1^iv^	76.55 (5)	Ba1^ii^—O4—Ba1^vi^	106.43 (5)
O4^iii^—Ba1—N1^iv^	87.26 (5)	C6—N1—Ba1^vii^	94.09 (12)
O3^iii^—Ba1—N1^iv^	66.27 (5)	C6—N1—H1A	108.9
O1^i^—Ba1—O1	73.03 (5)	Ba1^vii^—N1—H1A	123.8
O1W—Ba1—O1	155.90 (5)	C6—N1—H1B	110.7
O4^ii^—Ba1—O1	97.10 (4)	Ba1^vii^—N1—H1B	136.5
O2—Ba1—O1	45.23 (4)	H1A—N1—H1B	82.2
O4^iii^—Ba1—O1	119.31 (4)	O2—C1—O1	122.41 (18)
O3^iii^—Ba1—O1	136.85 (5)	O2—C1—C2	118.51 (17)
N1^iv^—Ba1—O1	73.66 (5)	O1—C1—C2	119.08 (17)
O1^i^—Ba1—C8^iii^	147.07 (5)	O2—C1—Ba1	56.41 (10)
O1W—Ba1—C8^iii^	67.76 (5)	O1—C1—Ba1	66.40 (11)
O4^ii^—Ba1—C8^iii^	90.03 (5)	C2—C1—Ba1	170.98 (14)
O2—Ba1—C8^iii^	93.92 (5)	C3—C2—C7	119.75 (18)
O4^iii^—Ba1—C8^iii^	23.28 (5)	C3—C2—C1	120.48 (17)
O3^iii^—Ba1—C8^iii^	22.89 (5)	C7—C2—C1	119.77 (17)
N1^iv^—Ba1—C8^iii^	78.98 (5)	C4—C3—C2	120.14 (18)
O1—Ba1—C8^iii^	134.77 (5)	C4—C3—H3	119.9
O1^i^—Ba1—C1	95.28 (5)	C2—C3—H3	119.9
O1W—Ba1—C1	156.97 (5)	C3—C4—C5	119.66 (17)
O4^ii^—Ba1—C1	85.89 (5)	C3—C4—C8	121.00 (17)
O2—Ba1—C1	22.36 (4)	C5—C4—C8	119.32 (17)
O4^iii^—Ba1—C1	96.91 (5)	C4—C5—C6	120.87 (17)
O3^iii^—Ba1—C1	125.09 (5)	C4—C5—H5	119.6
N1^iv^—Ba1—C1	75.35 (5)	C6—C5—H5	119.6
O1—Ba1—C1	22.96 (4)	C5—C6—C7	118.75 (17)
C8^iii^—Ba1—C1	115.07 (5)	C5—C6—N1	120.32 (17)
O1^i^—Ba1—C6^iv^	74.74 (5)	C7—C6—N1	120.73 (18)
O1W—Ba1—C6^iv^	115.84 (5)	C5—C6—Ba1^vii^	107.12 (13)
O4^ii^—Ba1—C6^iv^	172.98 (5)	C7—C6—Ba1^vii^	97.58 (12)
O2—Ba1—C6^iv^	95.71 (5)	N1—C6—Ba1^vii^	60.87 (10)
O4^iii^—Ba1—C6^iv^	107.94 (5)	C2—C7—C6	120.68 (18)
O3^iii^—Ba1—C6^iv^	74.39 (5)	C2—C7—H7	119.7
N1^iv^—Ba1—C6^iv^	25.04 (4)	C6—C7—H7	119.7
O1—Ba1—C6^iv^	76.13 (5)	O3—C8—O4	123.16 (18)
C8^iii^—Ba1—C6^iv^	93.49 (5)	O3—C8—C4	119.92 (18)
C1—Ba1—C6^iv^	87.11 (5)	O4—C8—C4	116.91 (17)
O1^i^—Ba1—Ba1^v^	141.47 (4)	O3—C8—Ba1^vi^	63.76 (11)
O1W—Ba1—Ba1^v^	70.99 (3)	O4—C8—Ba1^vi^	61.84 (10)
O4^ii^—Ba1—Ba1^v^	37.54 (3)	C4—C8—Ba1^vi^	162.79 (14)

Symmetry codes: (i) −*x*, −*y*+1, −*z*+1; (ii) −*x*, −*y*, −*z*+1; (iii) *x*, *y*+1, *z*−1; (iv) *x*, *y*+1, *z*; (v) −*x*, −*y*+1, −*z*; (vi) *x*, *y*−1, *z*+1; (vii) *x*, *y*−1, *z*.

### Hydrogen-bond geometry (Å, °)

**Table:**

*D*—H···*A*	*D*—H	H···*A*	*D*···*A*	*D*—H···*A*
O1W—H1WA···O3^i^	0.89	1.90	2.770 (2)	165
O1W—H1WB···O2^v^	0.83	1.95	2.770 (2)	167
N1—H1A···O2^viii^	0.96	2.16	3.067 (2)	157
N1—H1B···O4^ix^	0.99	2.19	3.176 (2)	175

Symmetry codes: (i) −*x*, −*y*+1, −*z*+1; (v) −*x*, −*y*+1, −*z*; (viii) −*x*+1, −*y*, −*z*; (ix) −*x*+1, −*y*−1, −*z*+1.

## References

[bb1] Brandenburg, K. (1999). *DIAMOND* Crystal Impact GbR, Bonn, Germany.

[bb2] Bruker (2001). *SADABS* Bruker AXS Inc., Madison, Wisconsin, USA.

[bb3] Bruker (2007). *APEX2* and *SAINT* Bruker AXS Inc., Madison, Wisconsin, USA.

[bb4] Kitagawa, S., Kitaura, R. & Noro, S. (2004). *Angew. Chem. Int. Ed.* **43**, 2334–2375. 10.1002/anie.200300610 15114565

[bb5] Kongshaug, K. O. & Fjellvåg, H. (2006). *Inorg. Chem.* **45**, 2424–2429. 10.1021/ic050662v 16529461

[bb6] Sheldrick, G. M. (2008). *Acta Cryst.* A**64**, 112–122. 10.1107/S0108767307043930 18156677

[bb7] Wu, C.-Y. & Lin, C.-H. (2010). *Acta Cryst.* E**66**, m1437. 10.1107/S1600536810040250 PMC3009009 21588860

[bb8] Zeng, R.-H., Fang, Z.-Q., Sun, F., Jiang, L.-S. & Tang, Y.-W. (2007). *Acta Cryst.* E**63**, m1813–m1814.

